# Agroforestry Spread of *Xylella fastidiosa* in Minorca: Vectors, Hosts, and Dynamics in Wild Olive Woods and Pastures

**DOI:** 10.3390/insects17101020

**Published:** 2026-10-02

**Authors:** Miquel Llompart, Josep Cifre, Diego Olmo, Andreu Juan, Jordi Sabaté

**Affiliations:** 1 Institut de Recerca i Tecnologia Agroalimentàries, Ctra. de Cabrils, km 2, 08348 Cabrils, Spain; jordi.sabate@irta.cat; 2 Departament d’Enginyeria Industrial i Construcció, Àrea d’Enginyeria Agroforestal, Universitat de les Illes Balears, Ctra. de Valldemossa, km 7.5, 07122 Palma, Spain; cifrepep@gmail.com; 3 Institut de Recerca i Formació Agroalimentària i Pesquera de les Illes Balears, 07009 Palma, Spain; dolmo@irfap.es; 4 Servei d’Agricultura, Conselleria d’Agricultura, Pesca i Medi Natural, 07006 Palma, Spain; ajuan@dgagric.caib.es

**Keywords:** Biosphere reserve, Mediterranean region, migration, *N. campestris*, phytoplasmas, *P. spumarius*, woody hosts, *X. fastidiosa* subsp. *multiplex*

## Abstract

Understanding the spread of *Xylella fastidiosa* (XF) in agroforestry systems is key to reducing its ecological and economic impact. In Minorca (Balearic Islands), the subspecies *multiplex* (ST81) was detected for the first time in 2017, mainly affecting wild olive (*Olea europaea* var. *sylvestris*), a key species in Mediterranean shrublands. This study analyzes XF dynamics and its insect vectors, *Philaenus spumarius* (PS) and *Neophilaenus campestris* (NC), over three years (2019–2021) in six agroecosystems. A total of 621 Auchenorrhyncha specimens were collected and analyzed by qPCR. XF was detected in 11.88% of PS but not in NC. PS was predominantly captured on herbaceous cover and *Pistacia lentiscus* and less often on wild olive and *Rhamnus alaternus.* Infection rates in PS peaked in summer and autumn, reaching over 50% in some months, especially on wild olives. XF was also confirmed in wild olive in all plots, with infection rates between 10% and 55%. No phytoplasmas were found. These results highlight the major role of PS in XF transmission in Minorca, with wild olive as a key reservoir. The findings stress the importance of vector behavior, host plants, and landscape structure in managing XF in Mediterranean agroforestry systems, indicating migration to woody hosts as a key factor.

## Simple Summary

*Xylella fastidiosa* (XF) is a plant-pathogenic bacterium that threatens crops and natural vegetation in many Mediterranean regions. In Minorca, one of the Balearic Islands, the bacterium affects wild olive, an abundant woody plant that is an important component of the island’s characteristic landscape. This study investigated how insect vectors, host plants, and seasonal changes influence the spread of XF in six agroforestry areas over three years. We found that *Philaenus spumarius* (PS), an insect capable of transmitting the bacterium between plants, was frequently infected, whereas *Neophilaenus campestris* (NC) showed no infection. PS was mainly found on herbaceous vegetation and *Pistacia lentiscus*, but infected individuals were particularly frequent on wild olive during some periods. The bacterium was also detected in wild olives in all the studied areas. These results indicate that PS and wild olive play important roles in the local spread of XF. Understanding the seasonal movement of this insect from herbaceous vegetation to woody plants may help to design more effective management strategies and reduce the spread of the disease in Mediterranean agroforestry landscapes.

## 1. Introduction

*Xylella fastidiosa* Wells et al. (XF) is a globally distributed Gram-negative bacterium that colonizes the xylem vessels of host plants and the foregut of potential insect vectors [1,2,3]. XF has a broad host range, infecting numerous plant species and inducing a wide range of diseases, with significant impacts on agriculture, forests, landscapes, and gardens. Currently, XF is reported to infect 713 species from more than 89 different botanical families [4]. In the Balearic Islands, XF is reported to infect 38 species [5]. In olive trees, it causes olive quick decline syndrome (OQDS), which consists of leaf scorch and desiccation of small terminal branches that rapidly spread to the rest of the canopy, producing a burned appearance, and leading to the rapid death of the tree [6].

XF is naturally transmitted by xylem-feeding insects belonging to the suborder Auchenorrhyncha [7], including spittlebugs (family Aphrophoridae), froghoppers (family Cercopidae), cicadas (family Cicadidae), and sharpshooters (family Cicadellidae, subfamily Cicadellinae), which act as potential vectors of the bacterium. In Europe, the confirmed insect vectors of XF include *Philaenus spumarius* (L.) (PS), *Neophilaenus campestris* (Fallén) (NC), and *Philaenus italosignus* Drosopoulos and Remane, all within the Aphrophoridae family [8,9]. Among these, PS is considered the main vector across European outbreaks, due to its polyphagy and widespread presence in various ecosystems. Despite being commonly present, PS was initially underestimated as a threat until its significant role in XF transmission was identified [10]. Regarding its life cycle, PS is a univoltine species that overwinters as eggs on herbaceous hosts, where the nymphs subsequently develop, whereas adults can feed on both herbaceous and woody plants [11].

In Europe, an outbreak of XF was identified in Apulia (southeastern Italy) in olive trees affected by OQDS, which first appeared suddenly in 2010 [12]. Subsequently, XF was reported in France (2015), Spain (2016), and Portugal (2019), with emerging and expanding outbreaks already occurring [4,13,14,15,16].

In the Balearic Islands, the first detection of XF occurred in Majorca in November 2016, identified in samples from sweet cherry trees in a nursery located in Manacor [17]. In 2017, the presence of XF was confirmed in Ibiza and Minorca (Balearic Islands, Spain) [18,19]. The subspecies identified in Minorca was *multiplex* (ST81) [20].

The vegetation of the Balearic Islands is typically Mediterranean, comprising associations of sclerophyllous and evergreen species. In Minorca, the tree vegetation is found in oak groves, pine forests, garrigue, and maquis. In areas with higher rainfall and cooler temperatures, *Quercus ilex* L. dominates, accompanied by an understory of *Arbutus unedo* L., *Erica arborea* L., *Erica scoparia* L. subsp. *scoparia*, and *Rhamnus alaternus* L., among others [21]. In drier areas, the garrigue is dominated by *Olea europaea* L. var. *sylvestris* (Mill.) Lehr. and *Chamaerops humilis* L., while in even more arid zones, it consists mainly of *Rosmarinus officinalis* L. and *Erica multiflora* L. In coastal areas, forests of *Juniperus phoenicea* L. subsp. *turbinata* (Guss.) Nyman are present, while rocky zones host resilient shrub communities adapted to poor soils.

The forested area of Minorca represents 52% of the total land area, with the *O. europaea* var. *sylvestris* garrigue covering 48% of the island’s forested land [22]. This vegetation type has historically coexisted with extensive grazing systems, primarily by cattle, shaping the landscape and contributing to its biodiversity.

One of the defining features of the Minorcan landscape is the mosaic of pastures, cultivated fields, and patches of natural vegetation. The ecological value of this landscape system as a promoter of biodiversity is well documented [23,24,25,26]. Among the natural vegetation patches, the *Prasio-Oleetum sylvestris* O. Bolòs et R. Mol. association, locally known as *ullastrar*, plays a fundamental role as the main natural asset of the region and one of the most representative plant communities of the island. It is most commonly found in the southeastern half of Minorca.

This Mediterranean maquis, dominated by *O. europaea* var. *sylvestris*, forms dense shrublands interspersed with species such as *Phillyrea* spp., *Pistacia lentiscus*, and *Smilax aspera* L. Other characteristic species include *Clematis cirrhosa* L., *Prasium majus* L., *Lonicera implexa* Ait., *Tamus communis* L., and *R. alaternus* [27].

In many cases, this plant community is enriched by nitrophilous species, as the shade provided by these woodland patches is highly valued by grazing livestock. Consequently, species such as *Urtica membranacea* Poiret, *Chenopodium murale* L., *Erodium chium* Willd., *Solanum nigrum* L., *Sonchus oleraceus* L., and *Sisymbrium officinale* Scop. are commonly found in these habitats [27].

The spread and expansion of XF in Minorca may pose a threat to the agroforestry system, natural ecosystems, endemic biodiversity, and the island’s landscapes. Several factors influence this spread, including vector behavior, host plant availability, and environmental conditions. The unique combination of shrublands and woodlands, along with grasslands, forage crops, and diverse agricultural practices, creates a complex local environment that facilitates the spread and establishment of XF.

The lack of curative treatments for XF, coupled with limited knowledge of vector biology and the mechanisms driving disease spread, underscores the critical importance of research into XF epidemiology. This study seeks to deepen our understanding of XF transmission and spread in Minorca, an island designated as a UNESCO Biosphere Reserve (MBR) on 7 October 1993, where XF-susceptible woodlands, pastures, landscape, and human economy are closely interdependent. Biosphere Reserves are designed to combine biodiversity conservation with socio-economic development, scientific and local knowledge, and dissemination [28], making the study of diseases such as XF particularly relevant. Additionally, comprehending XF epidemiology—including vector population dynamics, host plant interactions, and infection rates—is crucial for developing effective control strategies and elucidating broader patterns of pathogen dissemination.

## 2. Materials and Methods

### 2.1. Plot Location Management and Characteristics

This study was conducted on the island of Minorca from 2019 to 2021. Six sampling plots were selected across the island based on XF prevalence and spread, with support and guidance from local agricultural authorities (Table 1). The assessment focused particularly on the vulnerable agricultural area with higher prevalence in *Migjorn* (the southern region of Minorca). This area was characterized by a high prevalence of symptomatic *O. europaea* var. *sylvestris* individuals showing canopy decline, branch dieback, and other symptoms commonly associated with XF infection. Throughout the study area, agricultural and livestock activities were noted in the sampled plots. These plots featured a mosaic of pastures, cultivated fields, and patches of natural vegetation of *Prasio-Oleetum sylvestris.* Sampling was carried out within these vegetation patches. These patches, embedded within herbaceous crops and pasture areas, included a natural herbaceous understory. The herbaceous vegetation was subjected to standard management practices on the island, involving cattle grazing in March and April, followed by complete drying from the second half of June onwards. Following rainfall in late summer or early autumn, depending on the year, the herbaceous vegetation regrew. Each sampled patch included vegetation as described in Table 1, predominantly consisting of *O. europaea* var. *sylvestris* and *P. lentiscus*. The selected plots represented typical agroecosystems of the island, combining cultivated areas with semi-natural vegetation and pastureland.

Twenty *O. europaea* var. *sylvestris* specimens per plot were randomly selected among individuals showing symptoms compatible with XF infection and analyzed to assess their infection status and determine the XF infection rate (XFI). Phytoplasmas were also analyzed in insect vectors to rule out their potential involvement in the epidemiology of the observed symptoms in the study area.

### 2.2. Insect-Vector Sampling

Auchenorrhyncha species were surveyed to capture potential vectors of XF. The study primarily focused on xylem-feeding species as potential vectors of XF, while phloem-feeding species were secondarily included to provide a broader characterization of the Auchenorrhyncha community and to assess the possible presence of other phytopathogens, such as phytoplasmas.

Sampling was conducted bimonthly within the Aphrophoridae adult flight period. Although sampling was planned on a bimonthly basis, minor deviations occurred due to logistical constraints. Additional sampling events were conducted during some periods, whereas in 2021 sampling was limited to a single campaign due to COVID-19 restrictions and unfavorable climatic conditions. To ensure comparability among years, data were analyzed according to seasonal sampling periods. When more than one sampling event was conducted within the same season, seasonal values were calculated as the arithmetic mean of all sampling events performed during that season. Sampling started in May. Insects were captured using sweep netting. A sweep net with a circular opening of 30 cm in diameter was used throughout the study. On woody plants, sampling was performed using horizontal sweeping movements across the outer canopy. Captured insects were collected and examined after completing each sampling unit. A total of 40 m^2^ per host plant, plot, and sampling date was established. For shrubs and trees (specifically *O. europaea* var. *sylvestris*, *P. lentiscus*, *R. alaternus*, *Ficus carica* L., and *J. phoenicea* subsp. *turbinata*), the sampling unit consisted of 4 m^2^ per selected individual host plant. When a given plant species was represented by at least ten individuals within a plot, ten specimens were randomly selected for sampling (4 m^2^ specimen^−1^ × 10 specimens = 40 m^2^). In cases where fewer individuals were available, all present individuals were sampled, resulting in a reduced sampled canopy area. Since not all plant species were present in all plots and, in some plots, some plant species were represented by fewer than 10 individuals, the number of insects captured was divided by the total area sampled.

For herbaceous vegetation within the sampled vegetation patches, sweep netting was conducted linearly by walking approximately 20 linear meters (0.50 m × 20 m = 10 m^2^), with four replicates per plot. Collected specimens were classified following Ribaut [29], Della Giustina [30], and Holzinger et al. [31] and stored at −20 °C until analysis. Some specimens could not be identified to species level, particularly within Cicadellidae and Cixiidae, due to the lack of reliable external diagnostic characters, as species-level identification in these groups often requires examination of male genitalia.

### 2.3. DNA Extraction from Plants and Insects

DNA from plants and individual insects was extracted using the CTAB/Chloroform method, following the established European and Mediterranean Plant Protection Organization (EPPO) [32] protocols. For xylem feeders (Aphrophoridae), only the heads were extracted, whereas entire individuals of phloem feeders were fully crushed, both using a TissueLyser II Qiagen^®^ (QIAGEN GmbH, Hilden, Germany). The extracted DNA was resuspended in 50 µL of HPLC-grade water and stored at −20 °C. DNA was extracted from approximately 1 g of fresh plant tissue following the EPPO standard method [32] at the official Plant Health Laboratory (PHL).

### 2.4. Detection of X. fastidiosa Subspecies and Phytoplasmas in Plants and Insects

The XF detection in plant tissues and insect vectors was conducted using three different qPCR methods targeting different genomic regions of XF: Harper et al. [33] and Francis et al. [34], and Dupas et al. [35] for subspecies identification in a multiplex qPCR following EPPO [31]. Phytoplasmas were analyzed using the method of Christensen et al. [36] following EPPO [37]. Primers and probes were synthesized by Metabion^®^ (Metabion International AG, Planegg, Germany). All reactions were performed using a QuantStudio 3 system (Applied Biosystems^®^, Waltham, Massachusetts (MA), United States of America (USA)). The Harper qPCR detection method targets the Ribosome Maturation Factor *rimM* gene, while the Francis method targets the *HL* gene [38]. DNA from pure cultures of different XF subspecies from the Balearic Islands, provided by the official PHL, and HPLC-grade water were used as positive controls and negative controls, respectively. To exclude nonspecific amplifications and weak positives, only samples showing a Ct value below 35 cycles across all three methods were considered positive.

### 2.5. Statistical Analysis

All statistical analyses were conducted using IBM SPSS Statistics version 28.0 [39]. Statistical significance for all data was accepted at a *p*-value < 0.05 (α-level). The chi-square test (χ^2^) was applied to identify significant differences in the ratio between PS and NC (PS/NC) across sampling seasonal periods and years, as well as to detect significant differences in the frequency of XF-infected PS specimens captured across different plots, years, and host plant species. Furthermore, χ^2^ was used to assess differences in XF prevalence affecting *O. europaea* var. *sylvestris* across plots. For data exhibiting a normal distribution and homogeneity of variance, a factorial analysis of variance (ANOVA) was performed separately for PS and NC to assess significant differences in (i) the mean number of captures per season and (ii) population density across seasons, plots, and years. The Duncan test was used to evaluate statistical differences between means (*p* < 0.05), as indicated by the ANOVA. A generalized linear model (GLM) with a negative binomial distribution was applied to analyze the number of PS captures per host plant and the number of XF-infected PS adults per host plant. Fixed factors included in the model were plot, year, and host plant. Pearson’s correlation coefficient was used to assess the relationship between the number of PS individuals captured and the number of XF-infected PS adults, as well as the correlation between XF-infected PS adults and XF-infected host plants per plot.

## 3. Results

### 3.1. Prevalence of X. fastidiosa in Wild Olive Plants

XF was detected in wild *O. europaea* specimens across all six agroecosystems studied, confirming the presence of the bacterium and justifying the selection of the plots. *O. europaea* var. *sylvestris* specimens exhibited leaf scorch and desiccation of small terminal branches. The most affected showed a burned appearance of the entire canopy and wilting. The XFI in wild *O. europaea* specimens varied significantly between plots (χ^2^ = 11.927, *p* = 0.036). The XFI ranged from 10.00% in Plot 4 to 55.00% in Plot 6 (Table 2). Other woody plants in the plots, such as *P. lentiscus*, *J. phoenicea* subsp. *turbinata*, *R. alaternus*, and *Ficus carica*, showed no symptoms and had not been reported as positive on the island by the official PHL at the time of the study.

### 3.2. Aphrophoridae Distribution and Host Plant

Over the three-year period, more than 600 Auchenorrhyncha specimens were collected, identified, and analyzed. The total number of insects per year, along with their taxa and the qPCR detection results for XF, is reported in Table 3. The collected insects belonged to five families, with the Cicadellidae family being the most species-diverse. The Aphrophoridae family was the most represented, with 505 individuals collected, with only PS and NC species. No other species of xylem feeders were captured. Detailed information on host plant species, plot distribution, and phloem-feeding insects is provided below in this section and in Section 3.3.

In total, 446 PS and 59 NC were captured in all the geographical areas sampled, except in Plot 6, where no NC were present. The highest number of PS adults was recorded in 2020; however, it was also the year with the lowest number of NC captures. The PS/NC ratio differed significantly between sampling periods (χ^2^ = 79.679, *p* < 0.001) and between years (χ^2^ = 79.865, *p* < 0.001). Seasonal differences in capture numbers were assessed separately for each species by ANOVA, revealing significantly higher captures of PS in spring compared to summer and autumn (*p* < 0.05), whereas NC showed lower overall captures with no significant differences among seasons (Figure 1).

It is important to note that the ANOVA analysis revealed statistically significant differences between PS and NC, with the population density of PS being more than five times higher than that of NC (PS: 0.16 ± 0.032 adults m^−2^; NC: 0.03 ± 0.011 adults m^−2^; F = 22.693, d.f. = 1, *p* < 0.001). The population density of PS exhibited significant differences (*p* < 0.05) among plots and seasons but did not show significant differences among years. The population density of NC exhibited significant differences (*p* < 0.05) only among years, with 2021 being the year with the highest population density (0.14 ± 0.070 adults m^−2^). These differences in population density were evaluated using ANOVA. Differences in population density were recorded between PS and NC, although a similar seasonal trend was observed for both species throughout the study (Figure 2 and Figure 3). Adults of both species were collected from May onwards. PS adults reached a peak in spring and then exhibited a steady decline until the end of the season in November. However, a secondary increase in autumn was also observed, although less pronounced than the spring peak, as shown in Figure 2. In 2021, sampling was conducted only in autumn, when 58 PS adults were recorded. The results of the NC captures showed two peaks: one in spring and another in autumn, with the latter being higher than the spring peak. During the summer, NC captures were very low.

In the case of PS, all initial captures were made in the spontaneous herbaceous cover, with a peak occurring in the spring of 2019. By the end of spring, PS transited to shrub and tree species. During the summer months, they gathered mainly on *P. lentiscus*. On this species, we captured 122 and 67 adult PS individuals in June and July 2020, respectively (Figure 4). PS returned to the ground cover as herbaceous species began to regrow for mating and egg-laying, a phenomenon observed during the autumn season.

Adult PS were captured in all environments, including the spontaneous herbaceous cover, as well as on trees and bushes (Figure 4). The majority of adult PS individuals were captured on herbaceous species, with 229 PS adults collected, representing 51.69% of the total PS individuals captured. Among woody plants, *P. lentiscus* was the preferred host, accounting for 90.78% of PS adults collected on shrubs and wild woody plants (n = 217), while *O. europaea* accounted for 7.83%. A few individuals (n = 3) were collected on *R. alaternus*. Most NC captures occurred on the herbaceous cover (n = 55), except for three specimens collected on *O. europaea* and one on *P. lentiscus*. No NC adults were captured on *R. alaternus*.

### 3.3. Detection of X. fastidiosa and Phytoplasmas in the Potential Insect Vectors

Over the three-year period, a total of 621 Auchenorrhyncha specimens were analyzed for the presence of XF and phytoplasmas (Table 3). Among the 446 PS individuals analyzed, 53 (11.88%) tested positive for XF. In contrast, none of the 59 NC specimens tested positive for XF. Only XF subsp. *multiplex* was detected among the positive cases. Additionally, no phytoplasmas were detected in any of the captured Auchenorrhyncha.

Among the phloem-feeding leafhopper species within the family Cicadellidae, one individual of *Agallia* sp. tested positive for XF (1+/16), and one adult of *Hyalesthes* sp. (Cixiidae) tested positive for XF (1+/52). It is worth mentioning that, during the study, specimens of this species were very abundant in the canopies of *O. europaea* var. *sylvestris*. The XF-positive *Hyalesthes* sp. individual was collected on this host. Additionally, within the family Issidae, one adult of *Agalmatium flavescens* (Olivier, 1791) tested positive for XF (1+/23). This individual was also collected on *O. europaea* var. *sylvestris*. In contrast, the XF-positive *Agallia* sp. individual was collected on herbaceous cover. In general, all *Hyalesthes* sp. individuals were collected on *O. europaea* var. *sylvestris*, except for a few specimens on *P. lentiscus*. Most *Agallia* sp. individuals were collected on *P. lentiscus*, with only a small number on *O. europaea* var. *sylvestris* and herbaceous cover. Similarly, most Issidae individuals were collected on *O. europaea* var. *sylvestris*, with fewer specimens on *P. lentiscus*. On the other hand, within the family Flatidae, a specimen of the invasive *Metcalfa pruinosa* (Say) was captured, although it tested negative for XF. None of the few XF-positive phloem-feeding species showed a Ct value of qPCR below 32 cycles, indicating that all positive detections were associated with relatively low bacterial loads.

For PS, the occurrence of XF-positive individuals did not differ between years (χ^2^ = 0.684, *p* = 0.710). The first XF-positive individuals were detected in May. Results indicated significant differences among seasonal sampling periods (χ^2^ = 24.216, *p* < 0.001). The rate peaked in late summer and mid-autumn, coinciding with the strongest qPCR titers detected (Ct value < 30) (Figure 5). November 2020 recorded the highest percentage of XF positivity among all sampling months analyzed.

The XF bacterium was detected in PS collected at all sampling points. The observed differences in infection rates between insects captured on different plots were not statistically significant (χ^2^ = 3.496, *p* = 0.624). The infection rate PS ranged from 8.16% (Plot 6) to 19.10% (Plot 4) (Table 4). The number of infected insects detected (53+/446; 11.88%) was not consistent with the number of infected plants (47+/120; 39.17%). However, correlation analysis did not reveal a statistically significant relationship between the number of infected plants and the proportion of infected vectors (*p* > 0.05).

As previously mentioned, PS was only captured on three woody species and the spontaneous herbaceous cover. Table 5 shows the number of positives relative to the total analyzed per host plant and year. The herbaceous species compartment and *P. lentiscus* specimens recorded the highest number of PS captures and positive insects, with 24+/229 and 23+/197, respectively. Over the three years, the percentage of PS individuals positive for XF captured on *P. lentiscus* was higher than that recorded on the herbaceous cover, at 11.68% and 10.48%, respectively. However, these observed differences in the total number of positive insects per host plant were significantly influenced by host plant species according to the GLM (Table 5). The observed differences in the percentage of PS individuals positive for XF by host plant were also not statistically significant (χ^2^ = 7.150, *p* = 0.067). PS individuals captured on *R. alaternus* and *O. europaea* var. *sylvestris* exhibited similar levels of positivity for XF, at 33.33% and 29.41%, respectively (Table 5). The percentage of PS individuals positive for XF on *O. europaea* var. *sylvestris* reached 50% in September 2019 and July 2020. For *P. lentiscus*, the highest percentage of insect vectors of the bacterium was recorded in July across all years.

## 4. Discussion

Understanding the epidemiology of XF in natural and agroforestry systems is essential for assessing its potential impact and developing effective management strategies. Currently, XF represents a growing ecological, social, and economic threat to the Balearic Islands and other European regions affected by this phytopathogenic bacterium, compromising the viability of agroecosystems. In this context, the interaction between the pathogen, its vectors, and host plants plays a crucial role in determining the extent of disease spread. The results of this study highlight the importance of vector ecology, host plant susceptibility, and landscape structure in shaping XF transmission dynamics.

Our findings reinforce the crucial role of spittlebugs in the transmission of XF across Mediterranean Europe. Nevertheless, prior to the detection of XF in the region, research on the woody host plants of these vectors was limited, as PS was not initially regarded as a significant threat to crop productivity [40,41,42]. Understanding vector ecology and its relationship with host plants constitutes a fundamental step in studying the epidemiology of XF infection and implementing strategies to mitigate its detrimental effects. This study contributes to the growing body of knowledge on XF and its vectors in Europe, particularly regarding wild woody hosts in agroforestry systems, a key reservoir whose management is more complex than that of cultivated crops. This is especially relevant in Minorca, where these systems integrate into agricultural plots, forming a mosaic of grasslands, cultivated fields, and patches of natural vegetation in an MBR. In addition to these particularities in disease management on the island, it is essential to consider the presence of a specific strain, XF subspecies *multiplex* (ST81), which is characterized by a broad host range compared to other strains [16].

Only two species of xylem-sap feeders were captured, with 446 PS and 59 NC individuals, confirming the low diversity of potential XF vector species in the Balearic Islands and the preeminence of PS [43,44,45,46]. The number of spittlebugs captured varied throughout the year and across sampled areas, similar to observations in other studies [43]. The population densities were higher than those recorded by López [45] for Minorca, under the same sampling effort. However, the population densities observed in the present study were lower than those reported by Llompart et al. [43] in Ibiza during the same period and under the same sampling effort. During the summer months, the highest numbers of PS adults were recorded on *P. lentiscus*, suggesting that this species was the preferred woody plant during this period. This phenomenon is attributed to the fact that insects feeding on xylem sap are highly sensitive to plant turgor and xylem nutritional composition [47,48]. This pattern has also been observed in Majorca [44]. PS captures could occur simultaneously on herbaceous and woody hosts, although this host shift does not appear to follow a predetermined pattern. Insects may remain on herbaceous vegetation, jumping randomly to woody hosts, even during summer if the herbaceous vegetation is not completely dry. However, the absence of water stress in herbaceous vegetation does not prevent the transition to woody hosts, although it may be hypothesized to reduce its frequency. These findings underscore the importance of considering seasonal and habitat-specific behaviors when assessing vector management strategies. The low captures in *O. europaea* var. *sylvestris* could be attributed either to its low attractiveness to the vector or to plants showing advanced disease symptoms. In Majorca, during the same study period, low captures were also recorded in *O. europaea* var. *sylvestris* [44]. Studies conducted in California by Almeida et al. [49] with *Homalodisca coagulata* Say reported limited attraction to crops with high disease severity.

Among the analyzed phloem-feeding leafhopper species, the detection rates of XF were notably low, with all positive samples exhibiting weak signals, remaining below the threshold of 32 cycles. XF-positive individuals, including *Agallia* sp., *Hyalesthes* sp., and *Agalmatium flavescens*, were collected on *O. europaea* var. *sylvestris*, although the *Agallia* sp. individual was collected on herbaceous cover. The weak positive detections may be associated with recent feeding on infected wild olive trees, although the underlying mechanisms remain unclear. Similar low detection rates for *Hyalesthes* sp. and *Agalmatium flavescens* were reported in Majorca by Llompart et al. [44]. Ben Moussa et al. [50] also found *Euscelis lineolatus* Brullé. Similar low detection rates for *Hyalesthes* sp. and *Agalmatium flavescens* were reported in Majorca by Llompart et al. [44]. Ben Moussa et al. [50] also found *Euscelis lineolatus* Brullé and other Deltocephalinae positive for XF in PCR assays. However, the high abundance of *Hyalesthes* sp. and its known role as a vector of phytoplasmas make it a species potentially worth considering. However, no *Hyalesthes* sp. specimens tested positive for phytoplasmas, nor did any other captured Auchenorrhyncha. Although several phloem-feeding species were included in the sampling, phytoplasmas were not detected in this study. These results are consistent with those reported by Llompart et al. [44] during the same period on the island of Majorca, where no phytoplasmas were detected in any Auchenorrhyncha.

The results indicated that XF was widespread on the island, and approximately 12% (53+/446) of PS individuals were positive for the bacterium. Ben Moussa et al. [51] obtained a similar XF prevalence at a site located 500 m from the central outbreak in the Salento peninsula (Southern Italy), where 9% of adults were found positive. Similarly, a comparable prevalence was identified in Majorca (Spain) [44]. None of the NC individuals tested positive (0+/59). Similar findings were reported by the Generalitat Valenciana [52] for NC in 2022, with only 2 out of 1452 individuals testing positive for XF. The absence of NC individuals testing positive for XF could be explained by the abandonment of the agroforestry system during the summer, as well as a low preference for *O. europaea* var. *sylvestris*, the main host of XF on the island [16]. During this season, virtually no captures were recorded in the area, suggesting a displacement towards forested zones with the presence of *P. halepensis*, a non-host plant for XF [53]. Lopes et al. [54] and Morente et al. [55] reported that in central Spain, as well as Llompart et al. [43] in Ibiza, NC adults were abundant in *P. halepensis* during the summer. Therefore, it is possible that NC left the area without having visited any *XF*-positive plants. These results support the conclusion that PS is the primary vector species on the island. During the same study period, Llompart et al. [43,44] reached the same conclusion on the islands of Ibiza and Majorca.

The number of XF-carrying adults did not vary significantly between plots and years but did vary according to the host plant, as indicated by the GLM analysis. The number of positive individuals increased during the summer and autumn. As in the study by Saponari et al. [9], the highest prevalence rate of XF was recorded in insects captured in early November. Similarly to Ben Moussa et al. [50], the abundance of PS per plot was strongly positively correlated with the XF prevalence in the insects per plot (r = 0.691 **). The only subspecies detected during the study, both in insects and in *O. europaea* var. *sylvestris*, was *multiplex*. These results corroborate that, to date, it is the only subspecies present on the island.

The high proportion of *O. europaea* var. *sylvestris* specimens testing positive for XF confirms its key epidemiological role in the studied agroecosystem. Taken together, these patterns indicate that host plant identity plays a key role in shaping XF transmission dynamics in the studied system. The markedly higher infection rates observed in PS individuals captured on this host, particularly during certain months, compared with the lower overall infection rates detected in PS collected on *P. lentiscus*, suggest a more secondary role of this host in the local epidemiology. Despite the high number of PS individuals captured on *P. lentiscus*, this species has not tested positive for XF in routine analyses conducted by the official PHL, suggesting that it is unlikely to act as a relevant host in the study area. Furthermore, Llompart et al. [44] examined *P. lentiscus* specimens from areas surrounding the study plots in Majorca, where a high number of *P. spumarius* individuals were positive for XF; nevertheless, all analyzed *P. lentiscus* samples were negative.

In the Valencian Community (Spain), a total of 13,654 individuals were analyzed individually between 2017 and 2021, with the XF bacterium detected in PS (6.80%), NC (1.71%), and *Neophilaenus lineatus* (3.17%) [52]. In Salento (Italy), Saponari et al. [9] reported an XF infection rate of 25% (40+/160) in PS within olive agroecosystems. Our overall PS positivity rate falls within a similar range, considering the high variability between plots observed in this and other studies [47,56].

The positivity rate of PS captured on *P. lentiscus* hosts suggests high mobility of PS, with multiple acquisition events. Given that *P. lentiscus* has not tested positive, infected vectors captured on this species were likely infected during previous feeding on other host plants present in the study area, such as *O. europaea* var. *sylvestris* or other potential reservoirs. However, due to the limited number of captures on some host plants and the current uncertainties regarding acquisition efficiency and bacterial dynamics within insect vectors, the specific source of infection cannot be determined. Furthermore, although PS is known to retain XF after acquisition, the relative contribution of the last plant visited to infection status remains unclear and should be interpreted with caution. However, epidemiologically, this dispersion is highly significant, creating more reservoirs and transmission opportunities.

In conclusion, our findings confirm that PS is the primary vector species of XF in Minorca. From a management perspective, preventing the colonization of woody hosts by PS adults should be considered an important objective for limiting XF transmission. This approach could be particularly effective when combined with the control of PS during the nymphal stages, when populations are easier to monitor and control, and with inoculum reduction strategies aimed at reducing the availability of infection sources and, consequently, vector infection rates. In a control strategy based on vector migration and host-plant preferences, a push–pull strategy exploiting the preference of PS for *P. lentiscus* could be hypothesized, similar to the approach proposed for the control of Bois noir of grapevines, in which *Vitex agnus-castus*, a preferred host of *Hyalesthes obsoletus* (Signoret), was used as a trap plant to reduce vector populations in vineyards [57,58]. It is also important to consider the phenological stage of PS when managing herbaceous plants, as mowing or other operations can facilitate the movement of PS adults toward cultivated woody species. Based on the data collected, it was not possible to confirm a role for NC in the transmission of the bacterium.

The high XF infection rates of the dominant wild olive woods with PS populations alternating pastures, woods, and cultivated trees make the eradication of XF in Minorca currently unfeasible. These findings offer critical insights into the dynamics of XF dissemination across the Mediterranean region, contributing to a broader understanding of the epidemiological processes underlying its spread. Expanding our understanding of the ecological and epidemiological interactions between XF, its vectors, and host plants is crucial for devising sustainable approaches. These findings not only enhance our knowledge of XF dissemination in Minorca but also contribute to a broader understanding of its behavior within the agroforestry system of the Mediterranean basin. Continued research into the ecology and transmission dynamics of vectors will be essential for implementing effective and regionally adapted strategies to manage the challenges posed by XF’s persistent expansion throughout Mediterranean Europe.

## Figures and Tables

**Figure 1:**
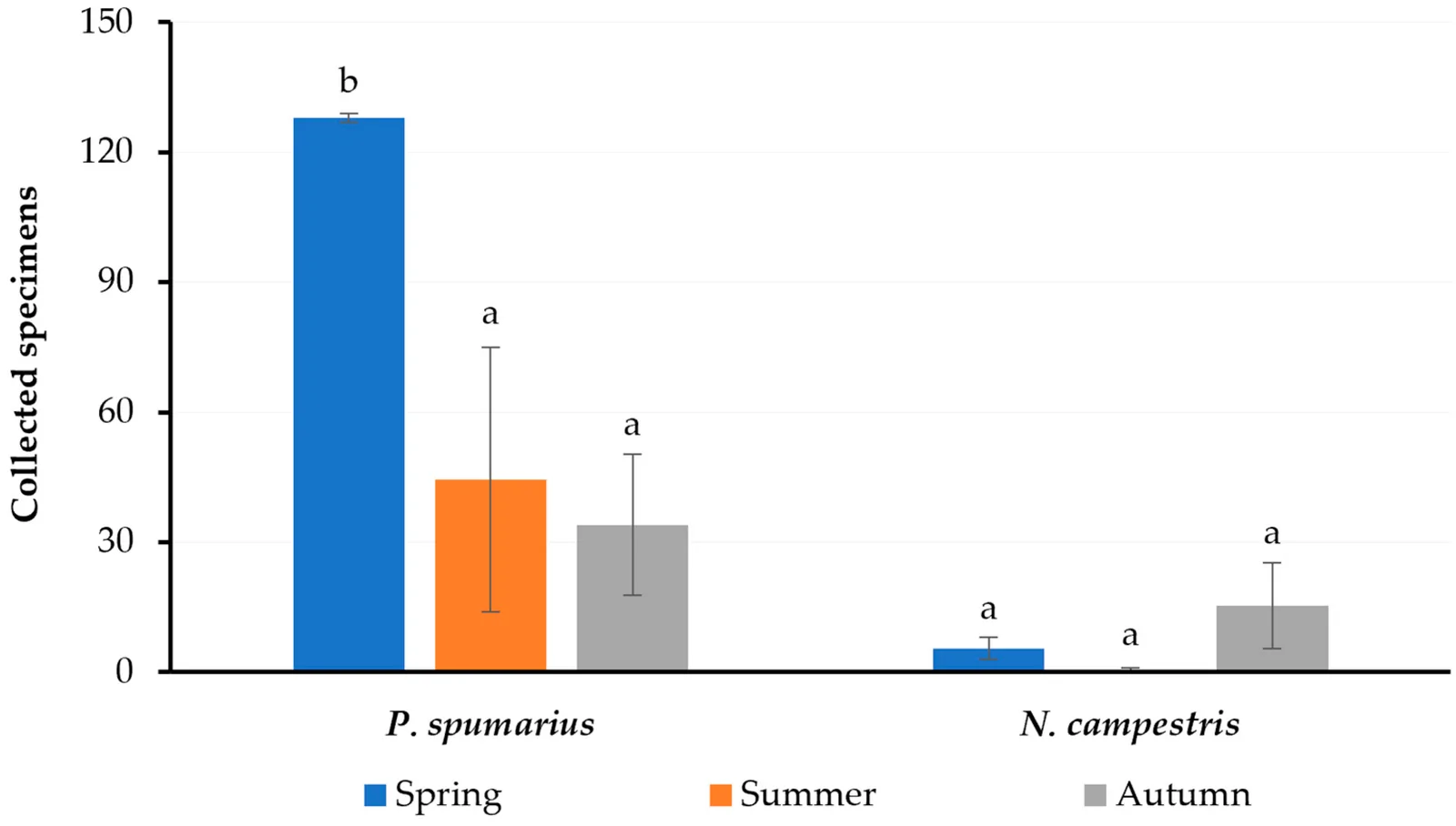
Mean seasonal number of collected *P. spumarius* and *N. campestris* adults captured (2019–2021). Data are presented as mean ± standard error (SE), calculated separately for each group. Different letters indicate significant differences (Duncan test, *p* < 0.05).

**Figure 2:**
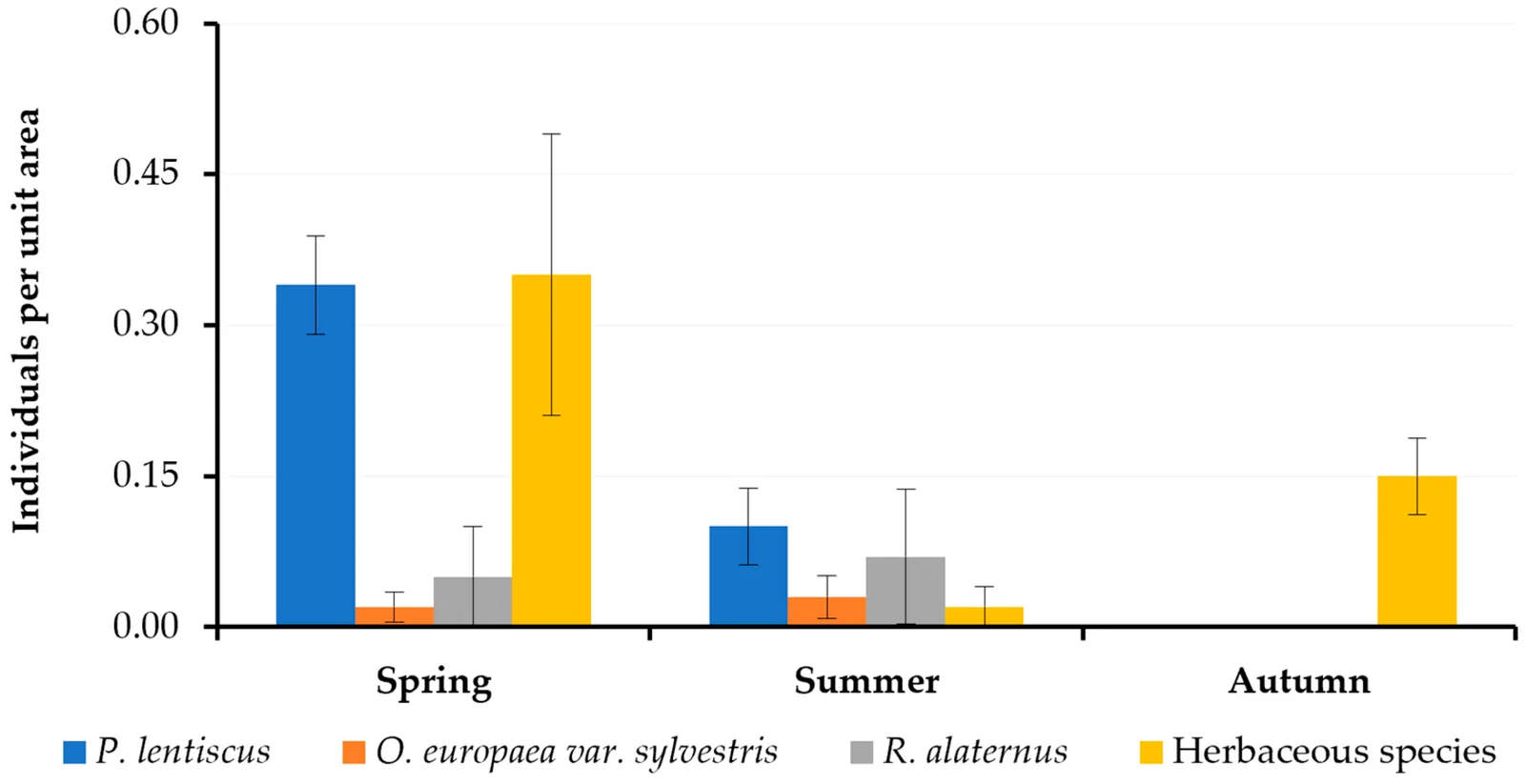
Mean density of *P. spumarius* adults collected on shrub and tree canopies and on herbaceous plants over the period 2019 to 2021. Data are presented as mean ± SE, calculated separately for each group.

**Figure 3:**
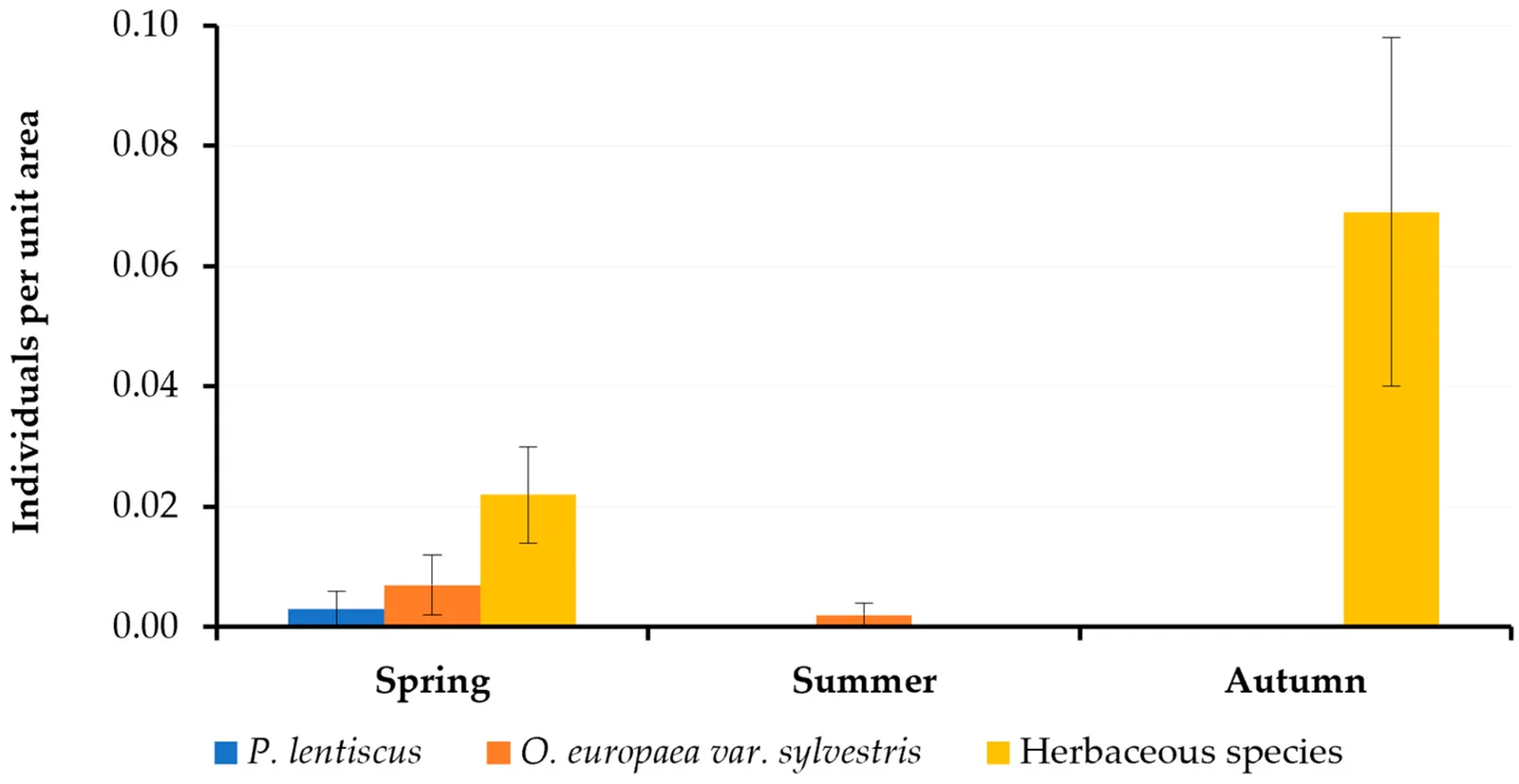
Mean density of *N. campestris* adults collected on shrub and tree canopies and on herbaceous plants over the period 2019 to 2021. Data are presented as mean ± SE, calculated separately for each group.

**Figure 4:**
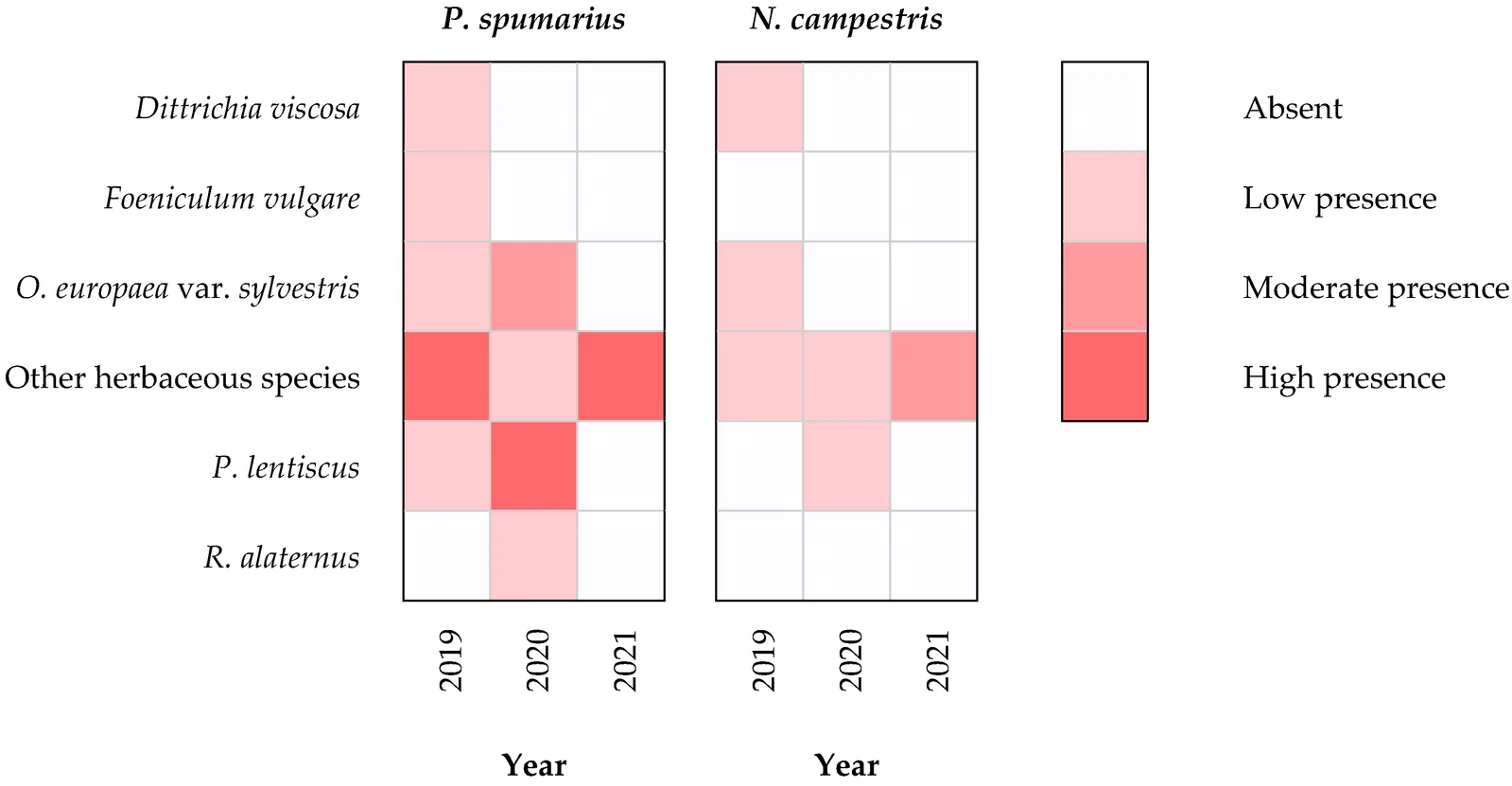
Host plant species of *P. spumarius* and *N. campestris* (2019–2021).

**Figure 5:**
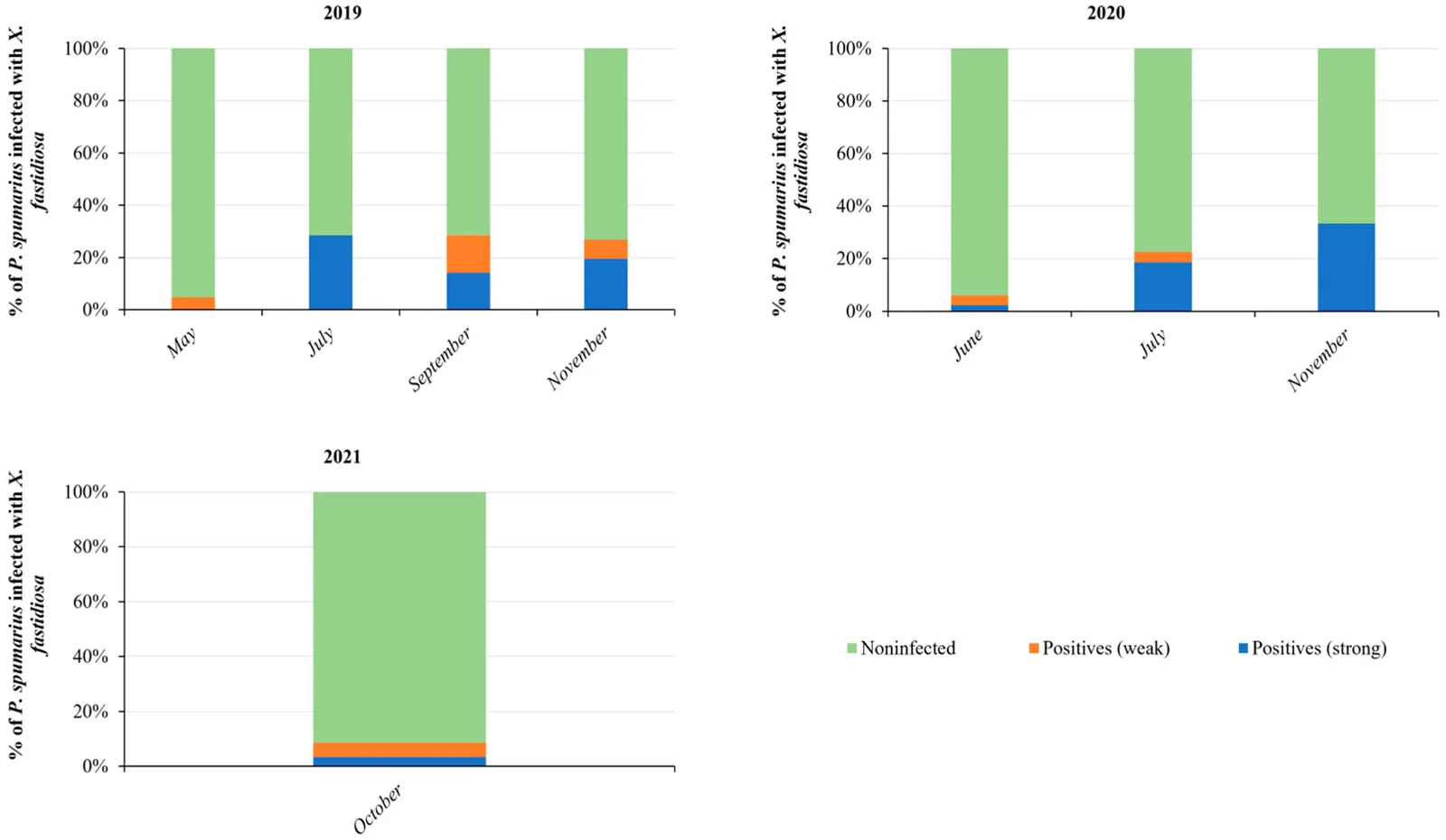
Percentage of *P. spumarius* individuals infected with ‘*X. fastidiosa*’. The results are presented per month of sampling and year. qPCR analysis by Harper et al. [33] and Francis et al. [34]. Strong positives (Ct value of qPCR < 30); weak positives (Ct value of qPCR 30–35).

**Table 1:** Plot location and woody vegetation of the sampled patches.

ID	Name	Location	Woody Vegetation
Plot 1	Sant Joan Gran	39°58′ N 03°52′ E	*Ficus carica*, *O. europaea* var. *sylvetsris*, and *P. lentiscus*
Plot 2	Binipati Nou	39°59′ N 03°53′ E	*J. phoenicea* subsp. *turbinata*, *O. europaea* var. *sylvetsris*, and *P. lentiscus*
Plot 3	Sa Muntanya	39°59′ N 03°57′ E	*O. europaea* var. *sylvetsris* and *P. lentiscus*
Plot 4	S’Aranjassa	39°58′ N 03°58′ E	*F. carica*, *O. europaea* var. *sylvetsris*, *P. lentiscus*, and *R. alaternus*
Plot 5	Torralbenc	39°53′ N 04°08′ E	*O. europaea* var. *sylvetsris*, *P. lentiscus*, and *R. alaternus*
Plot 6	Sant Vicent	39°52′ N 04°08′ E	*J. phoenicea* subsp. *turbinata*, *O. europaea* var. *sylvetsris*, and *P. lentiscus*

**Table 2:** Number and percentage of *O. europaea* var. *sylvestris* specimens infected with *X. fastidiosa* per plot. Positives (Ct value of qPCR < 35).

Title 2	qPCR Detection ^1^
Plot 1	10+/20 (50.00%)
Plot 2	10+/20 (50.00%)
Plot 3	8+/20 (40.00%)
Plot 4	2+/20 (10.00%)
Plot 5	6+/20 (30.00%)
Plot 6	11+/20 (55.00%)

^1^ qPCR analysis by Harper et al. [33] and Francis et al. [34]. Results are the number of positives (+)/total analyzed.

**Table 3:** Potential insect vector species collected during the 2019–2021 period. Identification of species that are vectors of *X. fastidiosa* and phytoplasma. Positives (Ct value of qPCR < 35).

Taxa	qPCR Detection ^1^				
	*X. fastidiosa*				Phytoplasma
	2019	2020	2021	Total	Total
**Aphrophoridae**					
*Neophilaenus campestris*	0+/21	0+/4	0+/34	0+/59 (0.00%)	0+/59 (0.00%)
*Philaenus spumarius*	21+/181	27+/207	5+/58	53+/446 (11.88%)	0+/446 (0.00%)
**Cicadellidae**					
*Agallia* sp.	1+/16	0+/0	0+/0	1+/16 (6.25%)	0+/16 (0.00%)
*Aphrodes* sp.	0+/6	0+/0	0+/0	0+/6 (0.00%)	0+/6 (0.00%)
*Artianus* sp.	0+/2	0+/0	0+/0	0+/2 (0.00%)	0+/2 (0.00%)
*Cicadula* sp.	0+/7	0+/0	0+/0	0+/7 (0.00%)	0+/7 (0.00%)
*Hardya* sp.	0+/7	0+/0	0+/0	0+/4 (0.00%)	0+/4 (0.00%)
*Psammotettix* sp.	0+/1	0+/0	0+/0	0+/1 (0.00%)	0+/1 (0.00%)
*Thamnotettix* sp.	0+/4	0+/0	0+/0	0+/4 (0.00%)	0+/4 (0.00%)
**Cixiidae**					
*Hyalesthes* sp.	1+/28	0+/24	0+/0	1+/52 (1.92%)	0+/52 (0.00%)
**Flatidae**					
*Metcalfa pruinosa*	0+/0	0+/1	0+/0	0+/1 (0.00%)	0+/1 (0.00%)
**Issidae**					
*Agalmatium flavescens*	1+/6	0+/17	0+/0	1+/23 (4.35%)	0+/23 (0.00%)

^1^ qPCR analysis by Harper et al. [33], Francis et al. [34], and Christensen et al. [36]. Results are the number of positives (+)/total analyzed.

**Table 4:** Number of annually collected *P. spumarius* captured within the study plots. Positive specimens (Ct value of qPCR < 35) are reported as both numbers and percentages.

Plot	qPCR Detection ^1^			
	2019	2020	2021	Total
Plot 1	3+/12 (25.00%)	1+/25 (4.00%)	1+/17 (5.88%)	4+/36 (11.11%)
Plot 2	1+/14 (7.14%)	4+/27 (14.81%)	2+/5 (40.00%)	7+/46 (15.22%)
Plot 3	3+/14 (21.43%)	6+/39 (15.38%)	1+/15 (6.67%)	10+/68 (14.71%)
Plot 4	6+/38 (15.79%)	10+/47 (21.28%)	1+/4 (25.00%)	17+/89 (19.10%)
Plot 5	4+/22 (18.18%)	4+/38 (10.53%)	0+/0 (0.00%)	8+/60 (13.33%)
Plot 6	2+/3 (66.67%)	2+/31 (6.45%)	0+/15 (0.00%)	4+/49 (8.16%)
Pearson correlation: percentage of PS-positive (+) adults and percentage of XF-infected host plants			r	−0.805
			*p*	0.055
Chi-square test: percentage of positives for XF			χ^2^	3.496
			*p*	0.624

^1^ qPCR analysis by Harper et al. [33] and Francis et al. [34]. Results are the number of positives (+)/total analyzed.

**Table 5:** Number and percentage of *P. spumarius* captured in Minorca vectors of *X. fastidiosa* per host plant and year (2019–2021). Positives (Ct value of qPCR < 35).

Host Plant Species of Adult *P. spumarius* Captured	qPCR Detection ^1^			
	2019	2020	2021	Total
Herbaceous species	17+/168 (10.12%)	2+/3 (66.66%)	5+/58 (8.62%)	24+/229 (10.48%)
*O. europaea* var. *sylvestris*	2+/5 (40.00%)	3+/12 (25.00%)	0+/0	5+/17 (29.41%)
*P. lentiscus*	2+/8 (25.00%)	21+/189 (11.11%)	0+/0	23+/197 (11.68%)
*R. alaternus*	0+/0	1+/3 (33.33%)	0+/0	1+/3 (33.33%)
GLM: total captures		AIC		510.226
		Deviance/df		1.120
		*p* (year)		0.213
		*p* (plot)		0.169
		*p* (host plant species)		<0.001
GLM: number of positives (+)		AIC		141.480
		Deviance/df		0.970
		*p* (year)		0.356
		*p* (plot)		0.275
		*p* (host plant species)		0.010
Pearson correlation: total captures and number of positives (+)		r		0.691
		*p*		<0.001
Chi-square test: percentage of positives for XF		χ^2^		7.150
		*p*		0.067

^1^ qPCR analysis by Harper et al. [33] and Francis et al. [34]. Results are the number of positives (+)/total analyzed.

## Data Availability

The original contributions presented in the study are included in the article; further inquiries can be directed to the corresponding author.
